# Response of rice production to rising CO_2_ and its adaptive cultivation strategies

**DOI:** 10.1016/j.fmre.2024.11.025

**Published:** 2024-12-12

**Authors:** Haiwei Zhang, Zihao Wang, Yuli Yan, Zihua Shi, Yu Jiang, Yanfeng Ding, Songhan Wang

**Affiliations:** a Jiangsu Collaborative Innovation Center for Modern Crop Production/Key Laboratory of Crop Physiology and Ecology in Southern China, College of Agriculture, Nanjing Agricultural University, Nanjing 210000, China; b Sanya Research Institute of Nanjing Agriculture University, Sanya 572024, China

**Keywords:** Rice yield, Rising CO_2_, CO_2_ fertilization effect, Physiological mechanism, Adaptive cultivation strategy

## Abstract

Rice is the staple food for more than half of the population in China. Therefore, enhancing the grain yield of rice is the core of ensuring food security. In recent decades, the increase of atmospheric carbon dioxide (CO_2_) concentration generally increases the photosynthetic rate of rice and then the rice yield. This phenomenon is generally termed the CO_2_ fertilization effect (CFE), which plays a pivotal role in sustaining global rice yield in the context of climate change. In order to accurately understand the impact of elevated CO_2_ on rice productivity and yield, this study first presents the mechanisms of CFE impacts on rice yield at the foliar and canopy levels. Elevated CO_2_ will promote the net photosynthetic rate, reduce the stomatal conductance, and thus increase the water use efficiency. Currently, at foliar, canopy, regional and global scales, controlled experiments, process-based models and statistical attribution models are the main approaches to estimate CFE. Based on these methods, at the leaf and canopy scales, elevated CO_2_ will lead to an increase in the number of panicles per unit area and the spikelet number per panicle of rice, finally resulting in the enhancement of grain yield. State-of-the-art crop models suggested that the global averaged CFE on rice yield is about 24% per 100 ppm increase in CO_2_, albeit with large differences between different models. Rice varieties, nutrient management, water regimes, planting density and temperature are the main factors that affect the CFE. Finally, based on the existing research basis and existing problems, we proposed several future research directions including the understanding of the mechanism of CFE from the perspective of rice roots, soil microbes, key genes and rice breeding, and also the upscaling of the results from site-level to regional- and global-level through the integration between experiments and models. By systematically reviewing the response of rice physiology and yield to elevated CO_2_ using multiple methods at various scales, this study will be beneficial for the formulation of adaptive cultivation strategies to enhance the rice yield and thus to sustain global food security in the context of climate change.

## 1 Introduction

Accounting for approximately 6% of the world’s water resources and 9% of the world’s arable land, China’s food supply should meet the requirements of > 22% of the world’s population. Therefore, food security has been identified as the top priority of China’s sustainable development strategy. Rice is the staple food for > 65% of China’s population [1], and its planting area ranks second and its annual total production ranks first across the globe. Therefore, sustained rice production is the core task in ensuring food security in China [2] A forecast suggests that, by 2030, China needs to increase rice production by 20% to meet the needs of its growing population [3]. However, due to the shortage of natural resources, limited arable land, low economic benefit of rice farming and frequent extreme climates, increasing rice production in China still faces dozens of challenges [4].

In recent decades, the speed of global climate change is becoming more severe. The continued increase in atmospheric carbon dioxide (CO_2_) concentrations is one of the most prominent phenomena of global change, and the main reason leading to global warming [5]. Since the age of the industrial revolution, with the rapid growth of the world’s population and the rapid development of the industrial and agricultural economy, the excessive use of fossil fuels and the change in land use have led to a sharp increase in atmospheric CO_2_ concentration [6]. The CO_2_ concentration has risen from 280 ppm at the time of the Industrial Revolution to about 419.3 ppm in 2023, with an increase of about 2.4 ppm per year [7]. Under current conditions, if no effective strategies are implemented, atmospheric CO_2_ concentrations are projected to continue rising steadily over the next few decades and are expected to reach 550 ppm by 2050 according to climate model projections [8].

Climate change is expected to increase surface and air temperatures, alter rainfall patterns, and increase the frequency of extreme weather events [9]. However, elevated CO_2_ generally has a positive effect on rice growth [10], despite its negative impacts on the global climate. CO_2_ in the atmosphere is a raw material for rice photosynthesis. Through the stomata and mesophyll tissues of rice leaves, CO_2_ in the air enters the chloroplast for photosynthetic activity. Therefore, the concentration of CO_2_ in the chloroplasts is reduced compared to the surrounding atmosphere [11]. Investigations have shown that under current atmospheric CO_2_ concentration (∼420 ppm), the intercellular CO_2_ concentration and chloroplast CO_2_ concentration of rice leaves were only about 280 ppm and 200 ppm, respectively, which are still lower than the CO_2_ saturation point of rice photosynthesis [12,13]. Therefore, increased atmospheric CO_2_ can increase the net photosynthetic rate of rice and the accumulation of dry matter, finally leading to an increase in grain yield [14]. This increase in photosynthetic rate, gross primary productivity (GPP), net primary productivity (NPP), biomass and grain yield due to enhanced atmospheric CO_2_ concentrations, is generally referred to as the “CO_2_ fertilization effect (CFE)” [15]. However, due to the CFE on rice yield is affected by several environmental conditions including temperature, water supply, soil moisture, etc., and also will be regulated by rice varieties, field nutrients and water management practices, the effect of enhanced CO_2_ on rice yield still shows large variations across different regions and different spatial scales. Therefore, an accurate investigation of the response of rice yield to elevated CO_2_ is urgently needed to accurately project global rice production in the context of climate change and is also the basis for formulating adaptation strategies to ensure global food security.

To this end, this review aims to comprehensively summarize the effects of elevated CO_2_ on rice physiology, productivity and grain yield at both the leaf, canopy, regional and global scales. The methods and approaches to assess the CFE at different scales and the current assessment results are also presented. Based on the response of global rice productivity to elevated CO_2_, we also summarize the current adaptive cultivation strategies to enhance the CFE on rice yield. Through this review, we aim to present the current advances of enhanced CO_2_ on rice productivity and grain yield and also provide useful clues for future studies on this topic.

## 2 Physiological basis of the CO_2_ fertilization effect

**Fig. 1:**
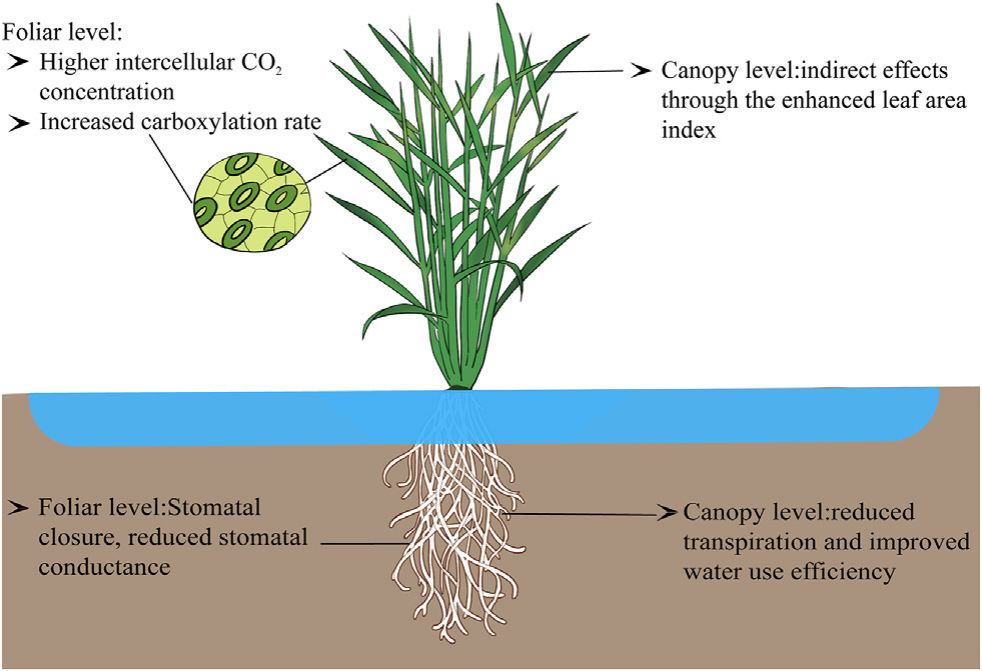
**Physiological basis of rising CO**_**2**_**impact on rice photosynthesis at the foliar and canopy levels.** Elevated CO_2_ can enhance intercellular CO_2_ levels in leaves, reduce photorespiration, boost photosynthetic rate, promote dry matter accumulation, and increase canopy leaf area index, optimizing photosynthetic efficiency at the canopy level. Additionally, stomatal closure reduces stomatal conductance, thereby enhancing water use efficiency in the canopy. (For interpretation of the references to colour in this figure legend, the reader is referred to the web version of this article.)

> 90% of crop yield is directly or indirectly derived from photosynthetic assimilates. For cultivated plants such as rice, the grain yield originates from the accumulation of carbohydrates in seeds, which mainly comes from the accumulation of photosynthesis during the growth period. Therefore, photosynthesis is the basis for crop economic yield and biological yield formation [16]. Atmospheric CO_2_ is an important raw material for crop growth. Crops assimilate the atmospheric CO_2_ through photosynthetic activity, producing organic matter and releasing oxygen (O_2_) at the same time [17]. Within the leaf, RuBP (Ribulose 1,5-bisphosphate) serves as the CO₂ receptor during photosynthetic activity, reacting with CO_2_ to produce two molecules of PGA (3-phosphoglyceric acid). These molecules then participate in a series of photochemical reactions, facilitated by light and various enzymes, to synthesize organic matter and regenerate RuBP, and finally complete the whole cycle of photosynthesis [18]. Therefore, the effect of atmosphere CO_2_ concentrations on rice photosynthesis generally originates from the following three ways, including the impacts on carboxylation rate, stomatal and mesophyll conductance at the foliar level, and also the impacts on leaf areas at the canopy level (Fig. 1).

### 2.1 Foliar level

Elevated CO_2_ generally enhances the photosynthetic rate at the foliar level [18,19]. There are two main reasons for this phenomenon (Fig. 1). On one hand, CO_2_ diffuses into the rice chloroplasts through the stomata and the mesophyll cells. Existing studies have shown that under the current CO_2_ level, the CO_2_ concentration within rice chloroplasts is only about 280 ppm. As a typical C_3_ crop, this level does not meet the requirements for rice ribulose-1,5-bisphosphate carboxylase (Rubisco) to achieve its maximum reaction rate. Consequently, the rate of rice photosynthetic is generally constrained by the CO_2_ concentration. Thus, an increase in atmosphere CO_2_ concentration enhances both the carboxylation and photosynthetic rates of rice [20].

On the other hand, in addition to serving as the CO₂ receptor facilitating the uptake of CO_2_ molecules for carboxylation, RuBP also serves as an oxidase, allowing O_2_ molecules to undergo oxidation, a process known as photorespiration [21]. Carboxylation increases the concentration of carboxylase substrate by absorbing CO_2_ and regenerating RuBP, whereas photorespiration consumes fixed carbon and is detrimental to carbohydrate accumulation [20]. At 25 °C, approximately 23% of the carbohydrates synthesized during photosynthesis are consumed by photorespiration. If the RuBP dedicated to photorespiration were redirected toward carboxylation, the carbon sequestration capacity of vegetation could increase by about 53% [22]. The ratio of CO_2_ to O_2_ within chloroplasts determines the predominance of these two reactions. An increase in CO_2_ concentration will boost the carboxylation rate while inhibiting photorespiration [18]. Therefore, the increased carboxylase activity and the decreased photorespiration caused by elevated CO_2_ are another reason for the increase in photosynthetic rate at the foliar level [23].

In addition to the net foliar photosynthetic rate, the water use efficiency at the foliar level will also be enhanced due to the increases in atmosphere CO_2_ (Fig. 1) [24]. When atmosphere CO_2_ increases, guard cells in rice leaves, possessing the inherent ability to detect changes in intercellular CO_2_ levels, will respond quickly by altering the activity of anion and cation channels in the cell membrane. This will lead to a reduction in cell membrane potential, a consequent decrease in stomatal aperture size, and finally result in a reduction of stomatal conductance [18]. The response of plant stomatal conductance to elevated CO_2_ depends on plant species [25], growth period [26], soil moisture conditions, and environmental conditions [27]. Previous studies suggest that the stomatal conductance of rice significantly decreases under elevated CO_2_ conditions, with an averag value of 25% [27]. This reduction ranges from 0% to 60%, depending on different CO_2_ field experiments, rice varieties, and growth phases [27]. The declines in stomatal conductance will increase stomatal resistance, reduce the water loss through foliar transpiration, and mitigate the effects of moderate drought stress on rice yield [28]. At the same time, the decrease in transpiration will reduce the flow of nutrients driven by transpiration, which may lead to the negative effect of CFE on rice due to insufficient nutrition. However, previous studies have found that elevated CO_2_ will not significantly reduce the absorption of nutrients by rice [29]. The possible reason is that, on one hand, the increase in CO_2_ will enhance the mineralization rate of soil nutrients [30],and on the other hand, the increase in CO_2_ will enhance the aboveground biomass [31]. Consequently, with the increases in foliar photosynthetic rate and the decreases in stomatal conductance, the water use efficiency of rice will be enhanced under elevated CO_2_. However, the interaction mechanism between transpiration reduction and nutrient absorption under elevated carbon dioxide needs further investigation.

Elevated CO_2_ also will down-regulate rice photosynthesis at the leaf level, i.e., photosynthetic acclimation [32], which possibly originates from two underlying mechanisms. The first is the source-sink regulation theory [33], i.e., the excessive accumulation of dry matter in the source organ cannot be transferred to the sink organ in time, or the insufficient sink capacity will produce a negative feedback on the photosynthetic rate. The other is the N-suppression hypothesis [34], which suggests that elevated CO_2_ would alter the balance of C and N pools, and that a decrease in leaf N content would inhibit the photosynthetic rate, ultimately limiting the response of rice to elevated CO_2_.

### 2.2 Canopy level

Upscaling from the foliar to the canopy level, the CO_2_ impacts on rice yield also depend on the rice leaf area (Fig. 1). The impacts of elevated CO_2_ on rice yield originate from the overall enhancement of biomass accumulation of the rice population, i.e., the increases in GPP at the canopy level. Based on the plant photosynthesis theory, GPP at the canopy level depends on both the net photosynthetic rate and the leaf areas. The effects of elevated CO_2_ on rice GPP thus consist of two components, of which one is the increased photosynthetic rate at the foliar level, and the other is the enhanced leaf area at the canopy level. Previous studies have shown that the leaf area index (LAI) acts as one of the controlling factors affecting the CFE values of GPP across the globe [35]. When atmosphere CO_2_ rises, the photosynthates and accumulation of dry matter in rice will be enhanced [36]. This accumulation, after allocation, will stimulate the development of rice tillers and leaves, and finally will increase the leaf area. Finally, the CFE on the net photosynthetic rate at the foliar level will be amplified to the canopy level through the increased leaf area [37].

## 3 Approaches to assess the CFE

**Fig. 2:**
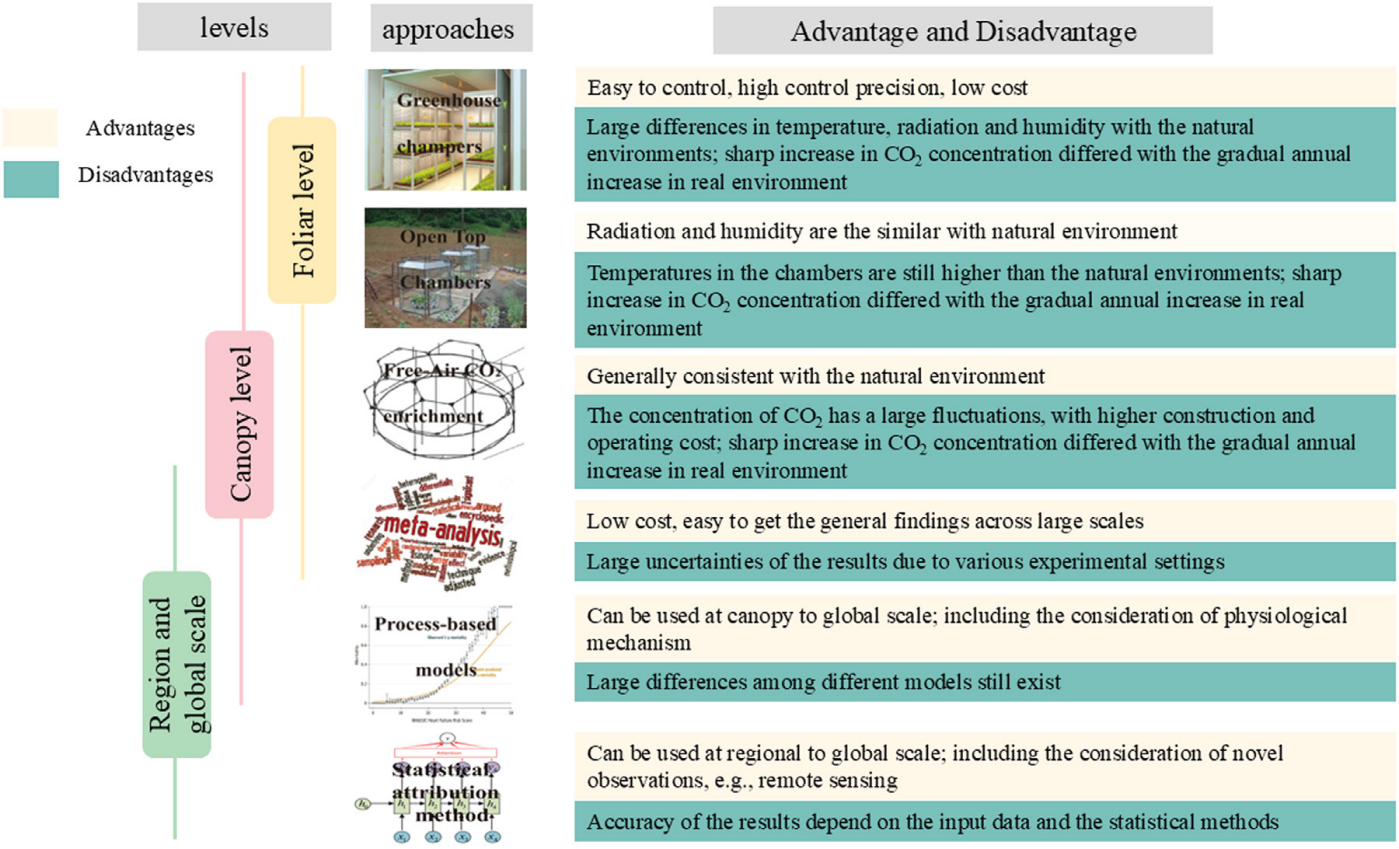
**Approaches to assess the CFE on rice productivity at different scales.** Different methods are used to evaluate the effects of CFE on rice productivity at different scales, and each method has its own advantages and disadvantages.

There are primarily six approaches to assess the CFE on rice productivity at different levels (Fig. 2):

(1) Greenhouse champers. CO_2_ enrichment experiments are widely used method to assess the impacts of CO_2_ fertilization on rice yield, by artificially enhancing the atmosphere CO_2_ concentrations and monitoring the physiological parameters and growth dynamics of plants [38]. In the early stage, the CO_2_ enrichment experiments are conducted in greenhouse chambers, utilizing intelligent greenhouse control systems to regulate the CO_2_ levels and ensuring a stable growth environment for plants [39,40], which can be used at the foliar and canopy levels. The advantages of greenhouse chambers are that they can precisely control the experimental conditions with high accuracy. They are also easy to replicate and generally have low operational costs. However, several limitations also exist. For example, the climate and environmental factors (temperature, radiation and humidity) in greenhouse chambers significantly differs from the natural environments. The limited space in greenhouse chambers also leads to a high ‘edge effect’, i.e., the plants planted at the edge of chambers generally experience different environmental conditions compared to those planted in the center. Changes in the microclimate of chambers and the ‘edge effect’ may lead to various responses of crop yield to elevated CO_2_ [41]. Additionally, the restriction of root growth due to limited space in greenhouses also affects the CO_2_ impacts [42,43]. A meta-analysis including various plant types suggested that the correlation coefficient of the major traits of plants in controlled and field environments was only 0.26 [44], indicating the significant limitation of greenhouse chambers.

(2) Open-top champers. As the upgrade of greenhouse chambers, the second type of controlled experiments utilized is the open-top chambers (OTC). Similar to greenhouse chambers, the advantages of OTCs is that their top is open to the atmosphere, making the atmosphere freely connected to the external environment. This design allows the radiation and precipitation in OTC to be the similar to those in natural environment [45,46]. Currently, OTCs are economical and manipulable controlled experiment that allows long-term studies to be carried out, and they can also be used at the foliar and canopy levels. However, some environmental conditions still have notable differences with the natural conditions. For instance, temperatures and humidity levels within OTCs tend to be higher than those in the natural environments.

(3) Free-air CO_2_ enrichment experiments. The state-of-the-art CO_2_ enrichment experiments are based on the Free-Air CO_2_ Enrichment (FACE) technique [47]. In the FACE experiments, CO_2_ is dispersed directly around plants via a circle of vertical pipes, with precise regulation an CO_2_ concentration through the auto-controlled system, maintaining other environmental variables such as temperature, radiation, precipitation and humidity unchanged [48]. The expansive and fully open-air setting of FACE experiments minimizes the impact of artificial conditions on the assessment of CO_2_ fertilization effects. Compared to greenhouse chambers and OTC, FACE experiments generally have large space and enable the studies of the CO_2_ fertilization effects across entire rice populations and throughout the full growth period, and also can be used to investigate the interactions between CO_2_ and other environmental factors on rice growth [10,49]. Therefore, FACE experiments are currently the most widely used approach to investigate the CO_2_ impacts on rice production at the foliar and canopy levels. Nevertheless, several limitations also exist for the FACE experiments, including the complex controlled system, high operational costs, and significant resource consumption.

(4) Meta-analysis. By synthesizing the findings from multiple previous independent studies on a similar topic, meta-analysis offers as an effective way to investigate the CO_2_ impacts on rice yield from the foliar, canopy to regional and global scales [50]. Using meta-analysis to assess the effects of elevated CO_2_ on rice productivity allows for a comprehensive summary that mitigates random errors associated with yearly variations and field tests, which has the advantages of identifying the general patterns. However, this method also has some limitations, e.g., it requires a substantial number of independent field-controlled experiments, and the accuracy of its findings largely relies on the reliability of the original datasets. The quality of the data included in the meta-analysis generally varies significantly. Thus the absence of uniform standards for data quality selection often results in substantial uncertainties of the outcomes from this method.

(5) Process-based models. Process-based models, including the crop models and the terrestrial ecosystem models, can be used to assess the CFE at regional and global scales. Evaluating the impact of CO_2_ on rice production using process-based models requires the simulation results of rice yield or GPP under scenarios of both constant and varying atmospheric CO_2_ concentrations [51]. The differences between these scenarios represent the CO_2_ impact on rice yield or GPP. For example, based on the widely used crop model simulations (AgMIP) or the terrestrial ecosystem model simulations (TRENDY), regional and global CO_2_ impacts on rice yield and GPP can be comprehensively assessed [52,53]. The advantages of process-based models are that they can be used to quantitatively assess the CO_2_ fertilization effect at different regions around the globe, which can alleviate the limitations of CO_2_ enrichment experiments. However, this approach also has its own shortcomings. For instance, the ecosystem processes of different models have large variation, so the effects of CO2 fertilization evaluated by various models generally differ remarkably [54]. Besides, the simulation of rice yield or GPP from models depends on some key specific assumptions, thus it possibly cannot fully represent the response of rice to increased CO_2_ under the changing climate and environmental conditions. For example, the dynamic vegetation model without a carbon-nitrogen interaction module will significantly overestimate the effect of CO_2_ fertilization [54]. Similarly, the existing models have not perfectly considered the phosphorus cycle, and therefore cannot fully reflect the limitation of phosphorus deficiency on the CO_2_ fertilization effect [55].

(6) Statistical attribution method. The statistical attribution model is a widely used approach to assess the CFE on rice yield and production. This model employs the statistical techniques to isolate the influence of rising CO_2_ on rice yield or GPP from the impacts of climate change and other environmental factors, using long-term datasets of rice yield or GPP as the input. In contrast to CO_2_ enrichment experiments and process-based models, the strength of statistical attribution method lies in its ability to assess the global distribution of CO_2_ fertilization effects straightforwardly, unencumbered by the limitations of FACE experiment setups or the various assumptions of process-based models. Moreover, with the aid of the long-term time series of rice yield or GPP data, the model can also be used to analyze the temporal trends in CO_2_ fertilization effect. Nevertheless, the statistical attribution model has some key limitions. The accuracy of the statistical attribution model depends on the quality of the input data, including both the response variables (e.g., yield and GPP) and independent variables (e.g., temperature and precipitation). Furthermore, the outcomes of this method are also largely influenced by the statistical model’s accuracy [55].

## 4 Current assessment results of the CFE on rice yield

### 4.1 Foliar and canopy level

**Fig. 3:**
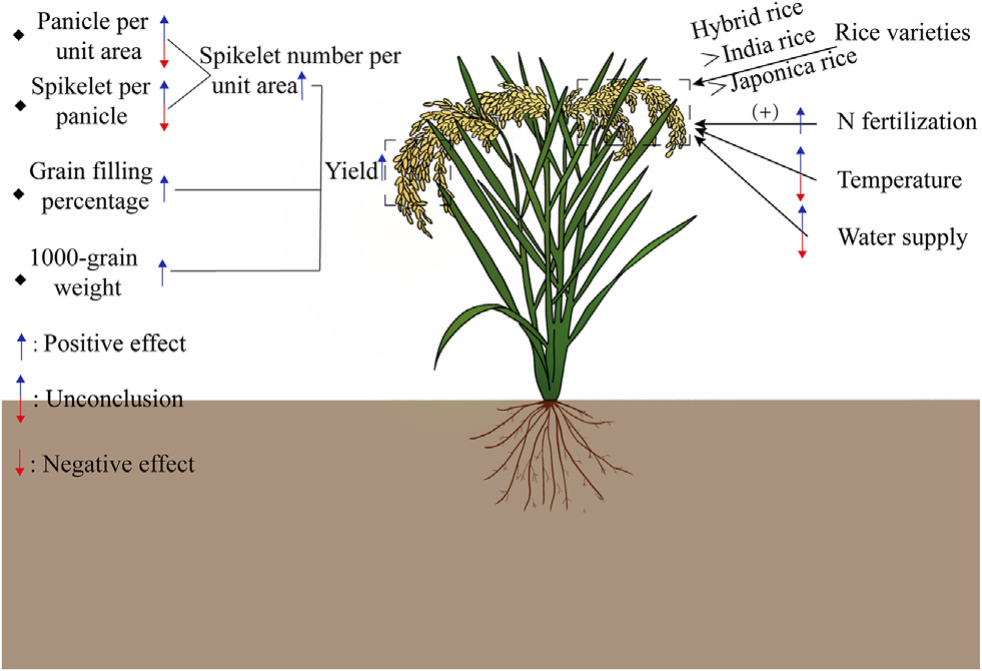
**Current assessment results of the rising CO**_**2**_**on rice production at the foliar and canopy levels.** Increasing CO_2_ can increase yield by increasing spikelet density, grain filling percentage and 1000-grain weight. Rice varieties, nitrogen fertilizer management, temperature and water supply will affect the CFE on rice yield. (+) means that increasing nitrogen application rate will enhance the CFE on rice yield.

The CO_2_ effect on rice yield includes its impact on four components: the number of effective panicles per unit area, spikelet number per panicle, grain filling percentage and 1000-grain weight (Fig. 3).

Rice yield is determined by the number of panicles per unit area, spikelet number per panicle, grain filling percentage and 1000-grain weight. Source-sink coordination is one of the physiological bases for high yield of rice. Previous studies have suggested that about 70% of the assimilates required for achieving rice grain filling come from the photosynthesis of functional leaves after heading, and about 30% come from non-structural carbohydrates (NSC) stored in stem sheath before heading [56]. Under normal CO_2_ levels, an increase in spikelet number (such as large panicle varieties) generally reduce the seed setting rate due to insufficient sources, which will ultimately lead to a reduction of grain yield [57]. On the contrary, under the condition of elevated CO_2_, the increase of spikelet number can delay the decrease of photosynthesis induced by the rapid accumulation of assimilates, and therefore the plenty of assimilates can ultimately promote the grain yield of rice.

(1) Effective panicle number per unit area. Hu et al. [58] found that among these yield components, the number of panicles per unit area exhibited the most significant response to increased CO_2_, with an average increase of 9.2%. Similarly, Lv et al. [59] found the similar conclusion through a regression model that the increase in the number of spikelets per panicle at elevated CO_2_ contributed the most compared to the other yield components. The possible reason for this phenomenon is that elevated CO_2_ can significantly increase the formation of rice tillers (11.41%), which is greater than the decrease in spikelet formation rate (−1.3%) [58].

(2) Spikelet number per panicle. Although it has also been reported that elevated CO_2_ significantly reduces effective tillering in rice [60,61] or generally shows no change [62,63], at present, there is a controversy about the effect of elevated CO_2_ on the number of spikelet per panicle [60,62,64]. Previous studies generally showed that elevated CO_2_ would enhance the rice sink capacity [[65], [66], [67]], represented by the number of spikelets per unit area, which is the product of the number of panicles and spikelets per panicle. Besides, Yang et al. [63] also highlighted that different rice varieties exhibit distinctly different CO_2_ effect, i.e., inbred Japonica rice generally benefits from an increased number of panicles, while hybrid rice generally benefits from a higher number of spikelet’s per panicle.

(3) Grain filling percentage and 1000-grain weight. The 1000-grain weight and grain filling percentage serve as the indicators of rice’s grain-filling capacity. Previous studies have suggested that elevated CO_2_ can enhance both the grain filling percentage and the 1000-grain weight in rice. However, in comparison to the number of panicles and the spikelet number per panicle, these two factors contribute less to yield increases under elevated CO_2_ conditions [25,68]. Through a three-year FACE experiment, Kim et al. [64] found that the CO_2_ impact on rice yield was primarily attributed to an increase in the spikelet number per unit area, with the 1000-grain weight playing a lesser role in yield augmentation. Similarly, based on a three-year FACE study in China, Yang et al. [69] also found that elevated CO_2_ generally led to the increases in the grain filling percentage (4.9%) and the 1000-grain weight (1.3%) of Wu Xiang Jing 14, albeit these increments were less significant than those observed in the spikelet number per unit area.

Together, these findings highlight that while various yield components collectively influence the rice yield under elevated CO_2_, the primary factors are the increases in the spikelet number per panicle and the number of effective panicles.

### 4.2 Regional and global levels

**Fig. 4:**
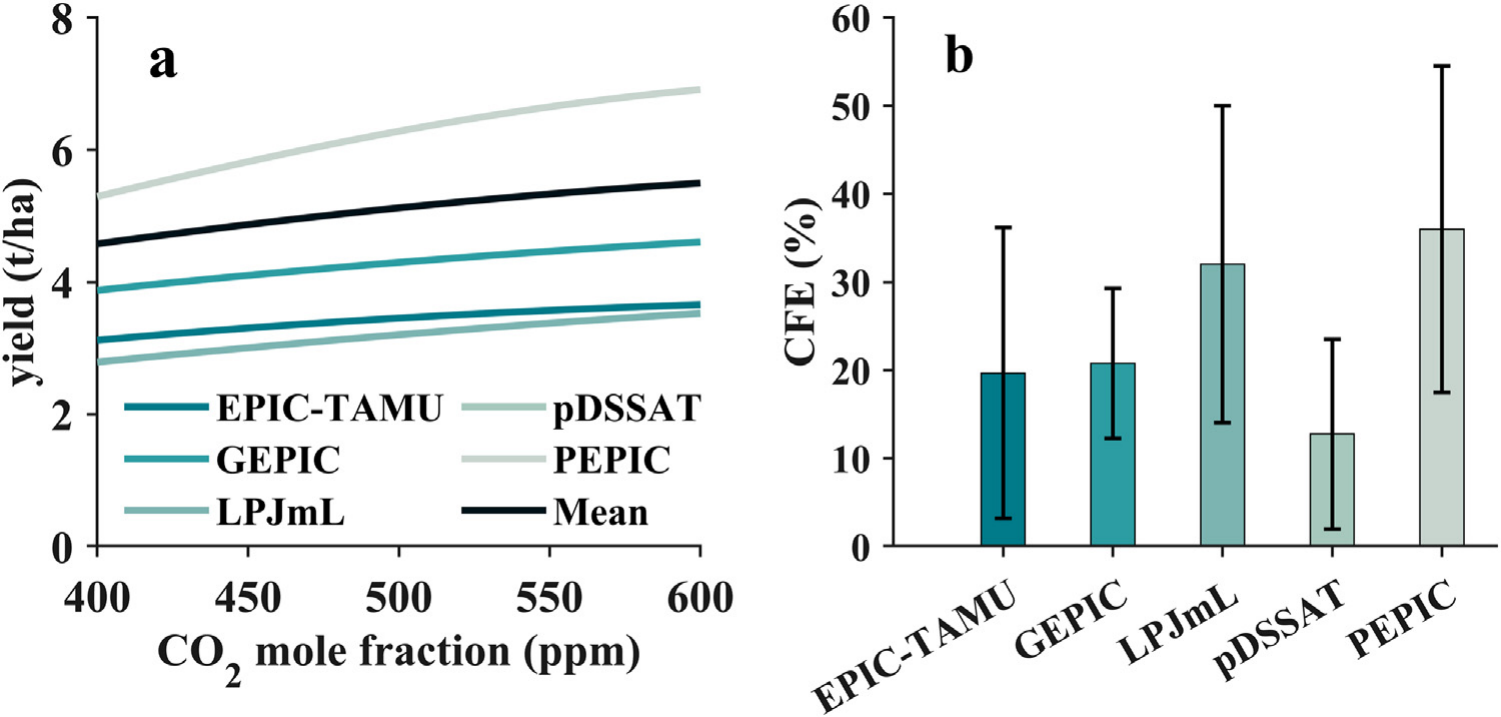
**Global assessment of rising CO**_**2**_**on rice yield from crop models.** a. Rice yield simulations of the five process-based crop models (EPIC-TAMU, GEPIC, LPJmL, pDSSAT, PEPIC) at the CO_2_ concentrations of 400–600 ppm. The multi-model values were also calculated as the average of the five crop models. b. The histogram of CFE estimates based on the five process-based crop models (EPIC-TAMU, GEPIC, LPJmL, pDSSAT, PEPIC) at the CO_2_ concentration increased from 400 to 600 ppm.

Currently, process-based models are the main approaches to assess the CO_2_ impacts on rice yield at regional and global levels. For example, based on the global gridded rice yield simulations at different scenarios from the GGCMI-2 models, the response of global rice yield to elevated CO_2_ can be evaluated. The rice yield simulated by pDSSAT model is the highest, which is about three times larger than that simulated by LPJmL model. More importantly, enhanced rice yields due to increased CO_2_ were observed for all of the crop models (Fig. 4a), albeit with a large difference between various models (Fig. 4b). When the CO_2_ concentrations increased from 400 ppm to 600 ppm, the rice yield enhancements simulated by the EPIC-TAMU, GEPIC, LPJmL, pDSSAT and PEPIC models are 17.2%, 18.9%, 26.5%, 12.5% and 30.6%, respectively. The PEPIC model predicted the highest increase rate of rice yield, while the pDSSAT model predicted the smallest. Averaging the results from various models, the rice yield would increase by about 20.1%, which is consistent with the results from previous field experiments [15]. Although we still cannot conclude which model is more accurate, the multi-model mean values of the CFE predictions from these models seems to be more agree with the results from field experiments. Therefore, large variations of the regional and global rice yield response to elevated CO_2_ still exist, due to the differences between various crop models.

### 4.3 Factors affect the CFE on rice yield

(1) Sub-species and varieties. The response of rice yield to elevated CO_2_ largely depends on the rice sub-species and varieties. Based on meta-analysis, previous studies suggest that the yield response to elevated CO_2_ for hybrid rice is approximately twice as high than that of conventional rice [58]. This significant difference is possibly due to the larger sink capacity and grain-filling ability of hybrid rice at elevated CO_2_. Further comparative analyses between two sub-species of conventional rice revealed that the yield increase for Indica rice is significantly higher than for Japonica rice [70]. Similarly, this disparity possibly because that Indica rice has a higher spikelet number per panicle and grain weight under elevated CO_2_ conditions, and also may due to the higher nitrogen use efficiency of Indica rice than Japonica rice [70]. Beyond the rice sub-species, the response of yield to elevated CO_2_ for different varieties also have large differences [72]. Although the differences in sink capability generally are suggested to be the possible reasons accounting for this phenomenon, several other processes including the divergent nutrient acquisition and use efficiency across different rice varieties also could be one of the reasons.

(2) Nitrogen fertilizer. Nitrogen (N) fertilizer is one of the important management practices to increase rice yield. Insufficient N fertilizer will limit rice tillering and reduce dry matter accumulation during the vegetative period, while excessive use of N fertilizer will result in lodging and aggravated pests and diseases. The current study indicates that low N levels could inhibit the positive effects of elevated CO_2_ on rice biomass, whereas high N levels were shown to significantly enhance dry matter accumulation during both vegetative and reproductive growth stages [60]. A global meta-analysis revealed that the impact of CO_2_ on rice yield enhancement becomes more notable with increasing N application rates (up to 30 g/m^2^) [58]. This effect is mainly due to the significant effects of N fertilizers on the percentage of grains filling, 1000-grain weight, and the number of effective panicles per unit area. In contrast, studies on the impact of excessive N application on rice yield under elevated CO_2_ conditions remains scarce, although some limited studies showed that applying excessive N fertilizer (> 30 g/m^2^) will also diminish the positive effects of elevated CO_2_ on rice yield [58].

(3) Temperature. Global meta-analysis has indicated that anthropogenic increases temperatures may negate the positive effect of elevated CO_2_ on rice yield, mainly due to the reduced grain filling percentage caused by increased temperature. Anthropogenic increases air temperature will affect rice photosynthesis and grain filling percentage, thereby diminishing the beneficial impacts of elevated CO_2_ on rice yield enhancement [15]. Moreover, anthropogenic increase temperatures will enhance the ratio of oxidation to carboxylation, leading to diminished photosynthesis in rice leaves [73]. Anthropogenic increasing temperatures will also shorten the rice growth period, reduce the time available for dry matter accumulation and transport, cause abnormalities in stamens and pistils, and lead to lower percentage of rice grain filling. However, some studies have shown that the increasing temperatures due to enhanced anthropogenic activity will increase the effect of CFE. The reason is that the atmospheric temperature is lower than the optimum temperature for rice growth, and increasing temperatures will stimulate rice growth. Therefore, increasing temperatures will generally reduce the positive effect of elevated CO_2_ concentration on rice yield, but some studies have shown that it will increase the effect of CFE, mainly depending on the atmospheric temperature and the optimum temperature for rice growth [58].

(4) Water regimes. Water regimes can influence CFE by affecting rice stomatal conductance and water use efficiency. Previous studies have shown that elevated CO_2_ will generally lead to reduced leaf stomatal conductance [58], reduced canopy transpiration [74], and increased water use efficiency [75]. Therefore, the consumption of water for rice production unit dry matter will be reduced [46], and lower water consumption in the early stages of rice can reserve more water for later seed filling. Such higher water reserves could sustain longer period for yield formation under drought conditions [76]. Similarly, Kimball et al. [77] found an average 19% increase in rice yield with elevated CO_2_ under wet conditions and an average 22% increase in yield under drought conditions, and thus they suggest the CFE is stronger under drought conditions. However, some studies have also shown that drought will reduce the CFE because elevated CO_2_ generally promotes rapid early growth of rice. This, on one hand, consumes more water, and insufficient soil moisture in the later stage will reduce the rice seed filling [78]. On the other hand, early rapid growth of rice produces more ineffective tillers, which do not form yields in the filling stage [79], and therefore reduces the CFE. In summary, although still has not reach a consensus, the effect of water regulation on the CFE may be influenced by the degree of drought and the period of rice fertility.

(5) Planting density. The planting density of rice critically influences the early growth and development of the rice population. An appropriate transplanting density is essential for the rational and efficient utilization of light energy and nutrients, establishing a robust foundation for individual development and population expansion of rice, which in turn significantly impacts the rice yield [80]. Liu et al. [62] investigated the yield formation of the hybrid rice variety Shanyou 63 under elevated CO_2_ conditions, and discovered that an optimal increase in planting spacing could maximize rice productivity in future high CO_2_ environments. Similarly, through FACE experiments, Yang et al. [63] clearly observed the existence of ‘edge effect’, which indicated that the properly increased planting density can enhance CFE on rice yield.

(6) Other factors. The CFE is affected by temperature, rainfall, soil nutrients and other factors. The climate conditions and nutrient supply in different regions are quite different. Therefore, the CFE in different regions also varies. Previous studies have shown that rice yield response to elevated CO_2_ shows clear differences across different regions, ranging from 5% to 17% per 100 ppm increase in CO_2_, with arid areas showing a larger response than humid areas [81].

The existing research platforms of CO_2_ elevation mainly include greenhouse, OTC and FACE. The CFE estimates from OTC and FACE experiments have some differences [68]. This may be because greenhouses and OTCs change the growth environment of plants, and the marginal effect are more obvious, which was more conducive to plant growth and exaggerate the CFE effects on rice yield [77].

## 5 Adaptive cultivation strategies

With the ever-increasing atmosphere CO_2_ concentrations, enhancing the CFE on rice yield through the adaptive cultivation strategies are of vital importance to secure global food security. The possible adaptive cultivation strategies may include the following four aspects (Table 1): ①cultivar selection and genetics improvement, ②nutrient management, ③water management and ④chemical regulating techniques.

**Table 1:** **Descriptions of the adaptive cultivation strategies**.

Adaptive cultivation strategies	Recommended actions	Mechanism
Cultivar selection and genetics improvement	Using varieties with large sink capability and high nutrient use efficiency	Alleviating the sink and nutrient limitation on CFE
Nutrient management	Postponing nitrogen application	Restricting ineffective tillering of rice and improving the CFE on photosynthesis at the reproductive stage
Water management	Alternate wetting and drying irrigation	Alleviating the constrains of reduced stomatal conductance on CFE and delaying rice root senescence
Chemical regulating techniques	Cytokinin, ABA inhibitor etc.	Increasing the sink capacity and alleviating the constrains of reduced stomatal conductance on CFE

In order to improve the CFE on rice yield, according to the mechanism of CFE, four aspects adaptive cultivation measures were put forward.

### 5.1 Variety selection and genetics improvement

Rice varieties are categorized into conventional and hybrid varieties, and can also be divided into indica and japonica sub-species. The responses of yield to elevated CO_2_ among different rice varieties show significant variations. Based on FACE experiments, previous studies have observed nearly doubled CO_2_ effects on the yield of hybrid rice than that of conventional rice [58]. Moreover, the CO_2_ effects on Indica rice are also significantly higher than thos on Japonica rice. These variations in yield response among different varieties and sub-species are suggested to be associated with spikelet number per panicle, with hybrid and Indica rice generally exhibit a higher spikelet number per panicle than that of Japonica rice. Similarly, a previous study conducted a correlation analysis of yield differences among different varieties using the FACE experiment, and found no significant differences in above-ground dry matter among different varieties under elevated CO_2_ [82]. Instead, the efficiency of producing economic yield per unit of dry matter is the key to determining rice yield. Furthermore, using various FACE experiments across Japan, previous studies have identified that panicle number per unit land area and the number of spikelets per panicle was the most important factors that accounting for the yield enhancement under elevated CO_2_ [83]. Altogether, these findings suggest that varieties with larger sink capacities generally exhibit a stronger response to CO_2_, and highlight the enhancement of the sink capability in rice possibly be a useful strategy to increase rice yield under elevated CO_2_ conditions.

In order to solve the problem of global food demand, it is necessary to develop rice varieties with stable and high yield under increasing CO_2_ concentrations. It is a useful strategy to introduce alleles that enhance sink capacity into conventional varieties [84,85]. For example, Hiroshi et al. [86] proposed a chromosome segment substitution line (CSSL) and a near isogenic line (NIL) to achieve the high spikelet numbers per panicle, demonstrating that using the allele genes could enhance rice yield by increasing sink capacity. Zhang et al. [87] utilized a novel hybrid assessment model, combining CERES-Rice with a machine learning model, to predict traits of future rice varieties suited to elevated CO_2_ conditions. They identified that the varieties with a medium fertility cycle, extended graining period, high photosynthetic capacity, numerous spikelets, and low stem height are generally the most promising varieties to achieve the increased sink expansion. Nevertheless, the improvement of the genetic techniques for enhancing the sink capability of rice is still needed in the future, in order to increase global rice yield in the context of climate change.

### 5.2 Nutrient management

The magnitude of CO_2_ fertilization is largely constrained by nutrient availability, with N being the most important one. As an essential major element for rice growth, N is the key component for formation amino acids, chlorophyll, nucleic acids and protein. The availability of N influences the rice photosynthesis by affecting the activity of carboxylase enzymes within the rice leaves [88,89]. Results from FACE experiments demonstrate that elevated CO_2_ would decrease the N content in rice leaves [90]. Under the situations of N limitation, foliar N will be reallocated to new leaves or leaves that entering early senescence. This re-distribution generally reduces the leaf N content, diminish both Rubisco content and activity, and subsequently limit the carboxylation rate. This reduction is the main reason for the photosynthetic acclimation observed in the reproductive stage of rice growth, highlighting the need to increase N fertilizer application in these stages.

Elevated CO_2_ generally increases ineffective tillering and reduces rice harvest index. Kim et al. [77] observed that while elevated CO_2_ levels enhance rice tillering, they also lead to an increase in ineffective tillering and a decrease in the effective spikelet rate. Thus, minimizing carbohydrate consumption by reducing ineffective tillers could also be a strategy to boost the rice yield under enhanced CO_2_.

The rational application of N fertilizer is very important for the improvement of rice economic yield and N use efficiency, which not only refers to the total amount of N fertilizers used but also to the timing and proportion of applications at different growth stages. The strategy of delaying N application, i.e., reducing early application and increasing it later in the growth cycle, can optimize the usage of N fertilizers. This management practice can curtail early tiller formation, thereby decreasing the maximum number of tillers and reducing ineffective tillers. It also can delay the leaf senescence in the later stages, enhance the photosynthetic capacity of leaves during the grain filling stage, and bolster the accumulation and transport of photosynthetic products. Ultimately, this approach can effectively increase rice yield [91]. Consequently, at elevated CO_2_ environments, delaying N application could enhance rice yield by minimizing ineffective tillering, enriching leaf N content during the reproductive period, and slow down the decline in carboxylation rate.

Beyond N, phosphorus (P) is also a crucial nutrient for the growth of rice. In environments with low P levels, rice exhibits a reduced rate of photosynthetic phosphorylation, leading to diminished photosynthetic products and impeded transport of these products [92]. The application of P fertilizers enhances the growth of rice roots and root hairs, boosts rice’s physiological activity and stress resistance, and facilitates the uptake of other nutrients such as N and potassium, thereby enhancing rice yield. Nevertheless, some previous studies have indicated that the excessive use of P fertilizer may hinder rice’s nutrient absorption [93]. These findings underscore the necessity of further research on the proper use of P fertilizers under elevated atmospheric CO_2_ conditions. Consequently, optimizing P fertilizers application may emerge as a pivotal strategy to support the growth and development of rice at elevated CO_2_.

### 5.3 Water management

Rice yield is determined by the interplay between dry matter accumulation and its transport. Accumulation during the vegetative growth phase forms the material foundation for high yield, whereas the distribution and transport of dry matter during the reproductive phase are crucial for yield outcomes. Cao et al. [94] observed that elevated CO_2_ increases the non-structural carbohydrates (NSC) content in stem sheaths before flowering, but does not significantly enhance the activities of enzymes responsible for converting sucrose to starch in the kernels post-flowering. Consequently, there is no notable increase in the 1000-grain weight, potentially due to hindered NSC transport to the kernels. In a FACE study, Kim et al. [64] examined the impact of elevated CO_2_ and N supply on rice yield in the temperate regions of Japan, and found that while elevated CO_2_ boosted rice yield, the rise in dry matter exceeded the yield increase. This discrepancy was attributed to a reduction in the rice harvest index caused by elevated CO_2_. Hu et al. [58] suggested that CO_2_ enhances dry matter accumulation in leaves, stems and spikelets, and increases dry matter distribution in roots and stems but decreases it in leaves. However, it does not alter the distribution in spikelets nor the harvest index, which reflects the efficiency of dry matter transfer to the seed.

Alternate wetting and drying (AWD) irrigation, a water-saving irrigation technique, is implemented in countries such as China, the Philippines, Thailand, and Vietnam. This method, characterized by mild dry-wet cycles, increases the root growth and development, enhance root physiological functions, and boost the root capacity to absorb water and nutrients. Consequently, it stimulates the shoot growth, increase the grain filling percentage, and improve the 1000-grain weight, all of which are beneficial for rice yield enhancement and quality improvement [95]. In addition, previous studies have revealed that moderate soil drought post-flowering in rice will elevate endogenous levels of abscisic acid (ABA). ABA not only plays a role in regulating rice senescence but also facilitates the translocation of NSC from the stem and sheath to seeds, thus enhancing the grain filling percentage and subsequently the grain weight and yield [96]. Nevertheless, the impact of AWD irrigation on rice yield formation under elevated CO_2_ conditions remains underexplored. It is hypothesized that AWD irrigation, in conjunction with elevated CO_2_, could enhance sink allocation and dry matter translocation to sinks, and consequently facilitates the enhancement in rice yield.

### 5.4 Chemical regulating techniques

Crop chemical regulation techniques, involving the use of natural plant hormones or synthetic plant growth regulators in crop production, aim to modify the endogenous hormone system of crops. This modification can alter the growth, development, and direction of crops towards desired outcomes [97].

The source-sink relationship is a critical metric for assessing rice population quality and its optimization is key to enhancing rice yield at elevated CO_2_. Chemical regulation plays an important role in regulating stomatal conductance and promoting photosynthesis during rice filling stage [98]. Besides, chemical regulation also can increase the number of spikelets per unit area of rice at the elevated CO_2_ conditions, which can finally improve the CFE of rice [99,100]. Li et al. [7] evaluated the impact of three chemical regulators on the source-sink dynamics of double-cropping rice in the southern part of Hunan Province, and found that aminoethyl hexanoate effectively increased the source strength, expanded the sink capacity, harmonized the source-sink relationship, and finally boosted the double-cropping rice yield by 13.8%. In elevated CO_2_ conditions, the discrepancy between actual and anticipated yields is often attributed to insufficient rice sink capacity, determined by spikelet density and average spikelet weight. Spikelet formation is crucially influenced by spikelet differentiation and degeneration processes, with achieving higher differentiation and lower degeneration rates essential for maximizing spikelet count per panicle. Han et al. [101] demonstrated that increased cytokinin (CTK) activity could enhance spikelet differentiation and spikelet count per panicle. Similarly, Zhang et al. [71] found that kinetin application could increase the yield of Indica rice varieties by boosting sink storage capacity, and also could enhance the CFE by 8% through FACE experiment. Zheng et al. [102] attributed the yield increase in wheat sprayed with 6-Benzylaminopurine before anthesis and found the compound’s ability to increase fertile floret numbers, reduce floret abortion rates, and significantly enhance the number of grains per spikelet and grain yield at maturity. Consequently, in elevated CO_2_ environments, rice yield can possibly be enhanced by applying these chemical substances through the enhanced sink strength.

## 6 Discussion and conclusion

Growing world population, increasing demand for cereals in animal feed, frequent extreme weather events caused by climate change, land loss due to urban expansion, and demand for bioenergy production are exerting increasing pressure on global agricultural productivity. In order to cope with the challenge of maintaining food security, agriculture must adapt quickly. While there are countless strategies that can do this, there is a growing recognition that excessive use of nutrients and irrigation does not provide a sustainable strategy for increasing crop yields, and using growing CO_2_ will be one of the most appropriate strategies. Therefore, the ultimate goal of increasing the CO_2_ response is to improve future rice yield and meet the growing food demand of humanity, by using the ever-increasing atmospheric CO_2_ concentrations with no economic cost.

Understanding the response of rice physiology and yield to increased CO_2_ is essential for adapting to future climate changes and maximizing the benefits of the CO_2_ fertilization effect to achieve stable and high rice yields. Previous studies on the impact of elevated CO_2_ on rice yield have been conducted at foliar, canopy, regional and global scales, evolving from greenhouse chambers to crop models. Previous studies have demonstrated that elevated CO_2_ enhances rice photosynthetic and carboxylase substrates, leading to higher net photosynthetic rates in leaves. The ‘source-sink’ regulation theory and the ‘N limitation hypothesis’ offer explanations for the photosynthetic acclimation observed under long-term elevated CO_2_ conditions. Increased CO_2_ reduces foliar stomatal conductance and transpiration water loss, and thereby improves the water use efficiency. Finally, carbon assimilation, dry matter accumulation and N absorption and utilization will be enhanced.

Experimental- and model-based results on rice yield responses to elevated CO_2_ vary significantly across different regions. Previous studies on rice yield responses to elevated CO_2_ and their physiological mechanisms have laid a theoretical foundation for leveraging CO_2_ fertilization effects to boost rice yield. However, there are huge differences in the response of different crops to CO_2_, and there are large genotypic differences in the response of rice yield to elevated CO_2_ [103,104]. there remain several aspects that need for improvement. Besides, in order to make full use of the CO_2_ fertilization effect, we need to combine improved varieties and integrate high-quality, high-yield and high-efficiency cultivation techniques. Several future directions are listed below: (1) The impacts of elevated CO_2_ on rice yield from the views of aboveground have made significant progress. However, the coupling relationship between rice root and soil is still relatively weak, especially the feedback of soil microorganisms and enzyme activities on long-term CO_2_ increase and its mechanism are still the key to research. (2) The genetic variation of rice yield to elevated CO_2_ has been quantified at the field scale, and the existing molecular breeding and genetics tools were used to elucidate the response mechanism of rice to elevated CO_2_ [103]. (3) Existing studies were conducted at different study sites, rice varieties, and management practices, and there are large differences between them. An integration of these results, by bridging the findings from various methods, is urgently needed at the regional to global scales. (4) Although large differences in yield response to elevated CO_2_ for different rice sub-species and varieties have been suggested, there is still a lack of molecular mechanism accounting for these differences. Therefore, it is necessary to strengthen the research on the molecular mechanism of yield difference and provide theoretical basis for rice breeding and variety selection. (5) Existing research about the response of rice yield to elevated CO_2_ mainly focuses on monoculture and rice-wheat rotation systems. There are large gaps in research on ratooning rice and double cropping systems, which have a long growth period and high land use efficiency, and is important in ensuring food security. (6) Adaptive cultivation measures to enhance the CFE are still limited. There have been many reports on the interaction between CO_2_ and nitrogen application, but the impacts of many other cultivation measures (such as N application ratio, N application period, N application method, water regimes etc.) on the CFE are still not fully explored.

## Declaration of competing interest

The authors declare that they have no conflicts of interest in this work.
